# New Appliances and Things Medical

**Published:** 1893-09-16

**Authors:** 


### NEW APPLIANCES AND THINGS MEDICAL.

### KUTNOW'S EFFERVESCENT CARLSBAD POWDER.

We have before us a sample of Kutnow's Improved Effervescent Carlsbad Powder. It is a fine white powder, and the advantages it possesses over other solid Carlsbad salts, is that it dissolves more easily than those which consist of large crystals; it is pleasant to the taste, and it effervesces on solution. Kutnow's preparation has been the subject of a recent very important legal decision. The Corporation of Carlsbad sought to restrain Mr. Kutnow from using as a trade mark a picture of the Hirschensprung or Deer-leap, a well-known object at Carlsbad. Mr. Justice North decided that the defendant might register this trade mark, and also the phrase "Kutnow's Improved Effervescent Carlsbad Powder." It was pointed out that even if the defendant should be unable to procure the Carlsbad salts direct from the spring at Carlsbad, the phrase Carlsbad salts had come to have such a wide significance that, like the phrase "Epsom salts," it no longer signified that the salts were obtained at the place which appeared in the name. Further the defendant in his title, used the words, "Kutnow, improved, effervescent, powder," all of which served to prevent confusion with any other Carlsbad salts.

